# Spatial and temporal trends in fin whale vocalizations recorded in the NE Pacific Ocean between 2003-2013

**DOI:** 10.1371/journal.pone.0186127

**Published:** 2017-10-26

**Authors:** Michelle J. Weirathmueller, Kathleen M. Stafford, William S. D. Wilcock, Rose S. Hilmo, Robert P. Dziak, Anne M. Tréhu

**Affiliations:** 1 School of Oceanography, University of Washington, Seattle, WA, United States of America; 2 Applied Physics Laboratory, University of Washington, Seattle, WA, United States of America; 3 National Oceanic and Atmospheric Administration (NOAA), Pacific Marine Environmental Laboratory, Newport, OR, United States of America; 4 College of Earth, Ocean, and Atmospheric Sciences, Oregon State University, Corvallis, OR, United States of America; Institute of Deep-sea Science and Engineering, Chinese Academy of Sciences, CHINA

## Abstract

In order to study the long-term stability of fin whale (*Balaenoptera physalus*) singing behavior, the frequency and inter-pulse interval of fin whale 20 Hz vocalizations were observed over 10 years from 2003–2013 from bottom mounted hydrophones and seismometers in the northeast Pacific Ocean. The instrument locations extended from 40°N to 48°N and 130°W to 125°W with water depths ranging from 1500–4000 m. The inter-pulse interval (IPI) of fin whale song sequences was observed to increase at a rate of 0.54 seconds/year over the decade of observation. During the same time period, peak frequency decreased at a rate of 0.17 Hz/year. Two primary call patterns were observed. During the earlier years, the more commonly observed pattern had a single frequency and single IPI. In later years, a doublet pattern emerged, with two dominant frequencies and IPIs. Many call sequences in the intervening years appeared to represent a transitional state between the two patterns. The overall trend was consistent across the entire geographical span, although some regional differences exist. Understanding changes in acoustic behavior over long time periods is needed to help establish whether acoustic characteristics can be used to help determine population identity in a widely distributed, difficult to study species such as the fin whale.

## Introduction

Large whales, particularly those that spend most of their lives well offshore and range widely throughout ocean basins, can be extremely difficult to study. Many baleen whale species, including fin whales (*Balaenoptera physalus*) are currently listed as *endangered* under the United States Endangered Species Act [1] and the IUCN Red List [2]; therefore understanding the population structure of these species is of particular importance for management and recovery efforts. Passive acoustic data have been used to successfully determine regions and seasons where different species of large whales occur, which has provided important information on the offshore distribution of endangered whales [3–5]. Passive acoustic data also hold the promise of identifying different populations of large whales based on stable acoustic signatures. For instance, the clear geographic variation in blue whale (*B*. *musculus*) songs has led some authors to propose that there are “acoustic populations” of blue whales and the signatures of each have been used to examine the population identity and geographic range of blue whales in all oceans [4–12].

Like blue whales, fin whales produce relatively simple, repeated signals that have appeared to be relatively invariant over time. The “20 Hz pulse” is the most commonly observed vocalization produced by fin whales and has been recorded throughout the world’s oceans [13–19]. The pulses are ~1-s-long and arranged into stereotyped sequences that can last for several hours [13]. Each is a downswept chirp [13] with a frequency range that, in the Pacific, can vary from ~40–25 Hz to ~20–15 Hz [18,20]. The interval between calls (inter-pulse interval, or IPI) is typically between 15–25 seconds with longer pauses of a few minutes that have been hypothesized to be surface intervals. The long series of 20 Hz calls are produced seasonally, usually from late fall until early spring [3,13,20–24]. The precise behavioral function of these calls is not known, although to date only male fin whales have been observed to produce these sounds, and their seasonality has been matched to the seasonality of fin whale reproduction; therefore it has been widely assumed that these long bouts have a reproductive function [25]. Because of this association with a reproductive display, and based on the repeated pattern of 20 Hz calls, the long series of these calls have been called “song” [9,11,23]. In the literature that describes song behavior in animals, the term “note” is used preferentially in the place of “call” due to the difference in behavioral context between the two terms, so we adopt “note” for the 20-Hz pulses that compose song from this point forward [26]. Where 20-Hz pulses are in irregular patterns, not song, they are referred to as “calls”.

There is evidence that some baleen whale species have song patterns that are related to specific geographic regions. Male humpback whales (*Megaptera novaeangliae*) from the same population sing the same song in the same year, and while that song changes during the season all males adopt the changes [27]. Song also changes from year to year, with striking changes observed over several years [28,29]. Blue, Bryde’s (*B*. *edeni)*, and minke (*B*. *acutorostrata*) whales also show evidence for geographic differences in song type but unlike humpback whales, their songs appear to be much simpler and are conserved over time in a given location [4,30–32]. Over time, the fundamental frequencies of some song notes of blue whales have decreased in frequency [33,34]. In some instances, this decrease appears to be quasi-continuous, whereas in others there is evidence of an annual “reset” in the fundamental frequency [34].

A number of studies have suggested that fin whale songs can be geographically distinct. The IPI of fin whales has been shown to vary in the Mediterranean Sea, and the North Pacific and North Atlantic Oceans [16,20,25,35–37]. In some regions, fin whales produce a higher frequency pulse associated with the 20 Hz note, and the frequency of that pulse has also been used to suggest geographic variation in fin whales around the Antarctic [38,39]. Additionally, song series may be composed of both a single pulse type or of two to three pulse types [20,40–42]. Because fin whales have been documented to change both the song note used and the IPI intra-annually [41,43], this study focuses on winter months (November through March) when songs are the predominant signals produced by this species [3,13,20,21]. Here we present 10 years of data from a single area in the NE Pacific Ocean and contemporaneous data from nearby locations to examine the IPI and frequency characteristics of fin whale song notes to determine how robust IPI and call characteristics are over a relatively long time scale and whether there is local geographic variation in these characteristics.

## Materials and methods

### Ethics statement

This study is based on passive recordings by ocean bottom seismometers and hydrophones deployed in the Northeast Pacific Ocean. The Keck Endeavour Seismic Network was deployed within the Canadian Endeavour Hydrothermal Vents Marine Protected Area (http://www.dfo-mpo.gc.ca/oceans/mpa-zpm/endeavour-eng.html) and permission for this work was obtained as part of the US Department of State obtaining authorization for this US research effort in Canadian waters. The NEPTUNE Canada cabled observatory was deployed by Ocean Networks Canada (http://www.oceannetworks.ca/observatories/pacific#northeast) who obtained all the necessary permits for its deployment and operation. All the other data were collected with temporary deployments of seafloor instruments in deep-water locations where no permits were required.

Fin whales are classified as endangered species. Opportunistic passive recordings of their vocalizations by seafloor instruments that were deployed to monitor earthquakes in waters of with depths of 1.5 km to 4 km do not interfere with their activities.

### Data

**Fig 1:**
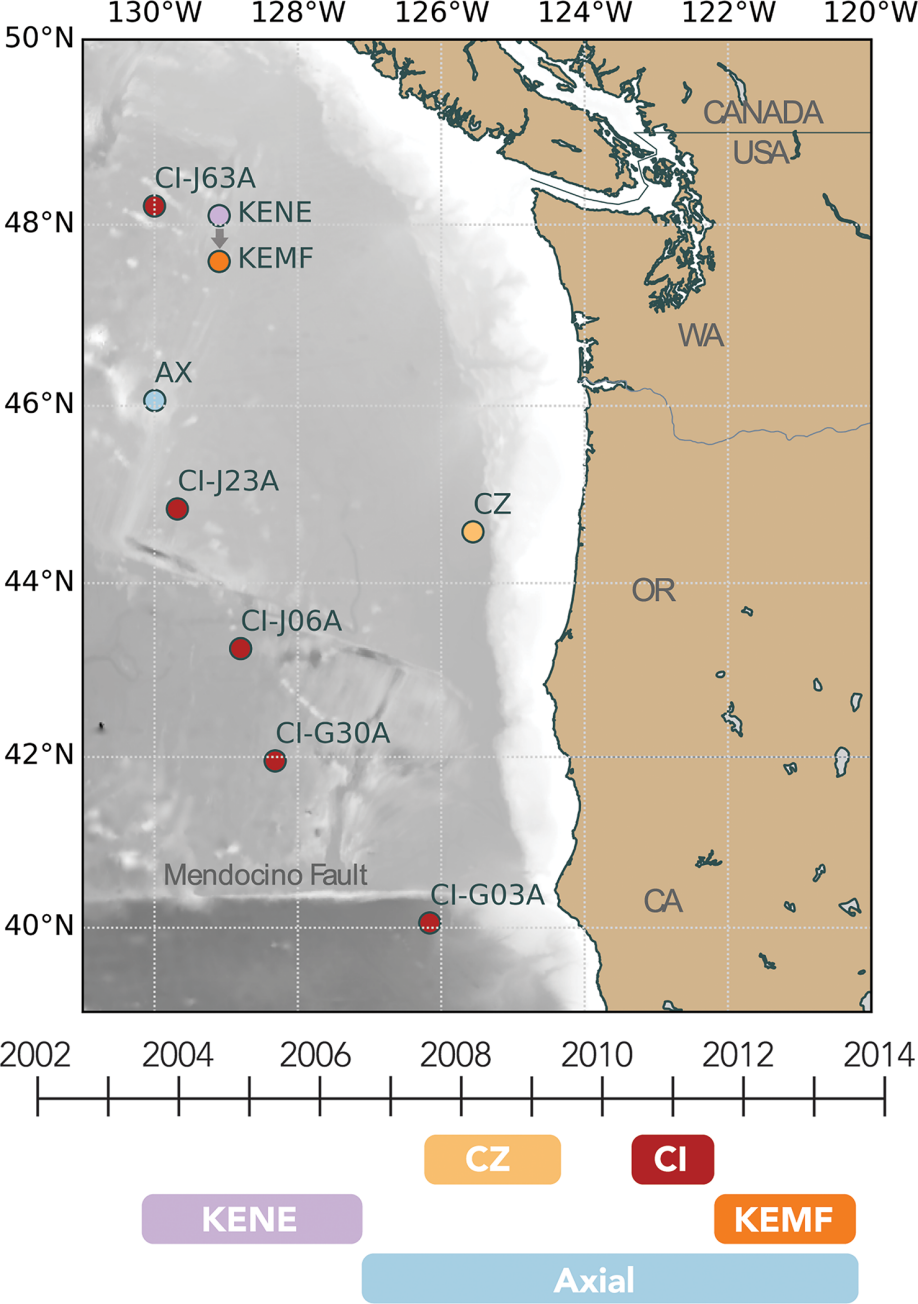
Regional map of the northeast Pacific Ocean showing locations of all instruments used in this study, along with a timeline indicating when they were deployed. At the scale of this map, KENE and KEMF are effectively co-located, but KENE is displayed slightly offset to the north for clarity (AX, Axial; CI, Cascadia Initiative; CZ, COLZA).

Data from ocean bottom hydrophones and seismometers deployed for five experiments were used in the analysis for this project. The locations of the instruments are listed in Table 1 and shown in Fig 1, along with the corresponding dataset timeline. With the exception of one instrument on Ocean Networks Canada cabled array, all the data were collected by autonomous instruments with individual deployments that extended from one summer to the next. Prior work has shown that seafloor recorders in the northeast Pacific will record fin whales with good signal to noise to a range of about 20 km [18,44], so each instrument samples fin whales vocalizing in a relatively small area.

**Table 1:** Instrument locations and recording times.

Experiment	Station	Sensor	Start Year	End Year	Latitude	Longitude	Depth, m	Sample Rate, Hz
NeMO	Axial	H	2006	2013	45.96°N	130.01°W	1550	100
KE	KENE	S	2003	2006	47.97°N	129.06°W	2330	128
ONC	KEMF	S	2011	2013	47.95°N	129.10°W	2205	100
COLZA	OBS01	S	2007	2009	44.58°N	125.56°W	2880	100
CI	J63A	S	2011	2012	48.21°N	130.00°W	2880	50
	J23A	S	2011	2012	44.84°N	129.68°W	2660	50
	J06A	S	2011	2012	43.25°N	128.80°W	3220	50
	G30A	S	2011	2012	41.96°N	128.32°W	3120	50
	G03A	S	2011	2012	40.06°N	126.16°W	4110	50

NeMO, New Millenium Observatory; KE, Keck Endeavour; ONC, Ocean Networks Canada; COLZA, Central Oregon Locked Zone Array; CI, Cascadia Initiative; H, Hydrophone; S, Seismometer.

The longest continuous time series is from an ocean bottom hydrophone (OBH) located at Axial Seamount near 46°N, 130°W in a water depth of 1550 m as part of the NOAA Pacific Marine and Environmental Laboratory’s New Millennium Observatory (NeMO) experiment. We used data collected on the Axial instrument over a duration of 7 years, from 2006–2013, recorded at a sample rate of 100 Hz. This dataset was used to examine long time-scale variation in fin whale calling behavior at a single location.

The other instruments used in the study were all ocean bottom seismometers (OBS), which are designed to measure ground velocity caused by earthquakes. These instruments have three seismometer channels: one vertical and two horizontal. They also often include a pressure sensor as a fourth channel but for all the data used in this study, the fourth channel is a differential pressure gauge [45] which is not sensitive to signals above ~10 Hz and thus not useful for studying fin whales. Only the vertical seismometer channel was used in this analysis because it is the most reliable seismometer channel to analyze for signals propagating through the water column. These instruments were used to extend the time series to 10 years and to search for geographic variations between different sites in the same year.

Data from two experiments on at the Endeavour segment of the Juan de Fuca Ridge, near 48°N, 129°W at a water depth of ~2250 m, were used in this study. The KENE instrument was deployed between 2003–2006 as part of an 8-station seismic experiment [46] and sampled at 128 Hz. The KEMF instrument is part of the Ocean Networks Canada Neptune cabled observatory and is located approximately 4 km to the southwest of the KENE instrument site. The sample rate was 100 Hz and we used data from 2010–2012.

We also analyzed data from a single instrument at the base of the Oregon continental slope that was deployed as part of the Central Oregon Locked Zone Array (COLZA) experiment [47]. This instrument was deployed from 2007–2009 and recorded at a sample rate of 100 Hz.

Finally, we used a subset of five OBSs from the Cascadia Initiative (CI) experiment, which is large seismic and geodetic experiment that was deployed from 2011–2015 [48]. We selected instruments deployed from 2011–2012 along the western periphery of the array, extending from 40°N to 48°N (Table 1). This allowed us to monitor the temporal patterns of calls over a north-south geographic span of nearly 1000 km during a time period contemporaneous with one year of the Axial dataset. The instrument depths varied from 2660 m to 4110 m and the sample rate was 50 Hz for all instruments.

### Automatic detection of calls

**Fig 2:**
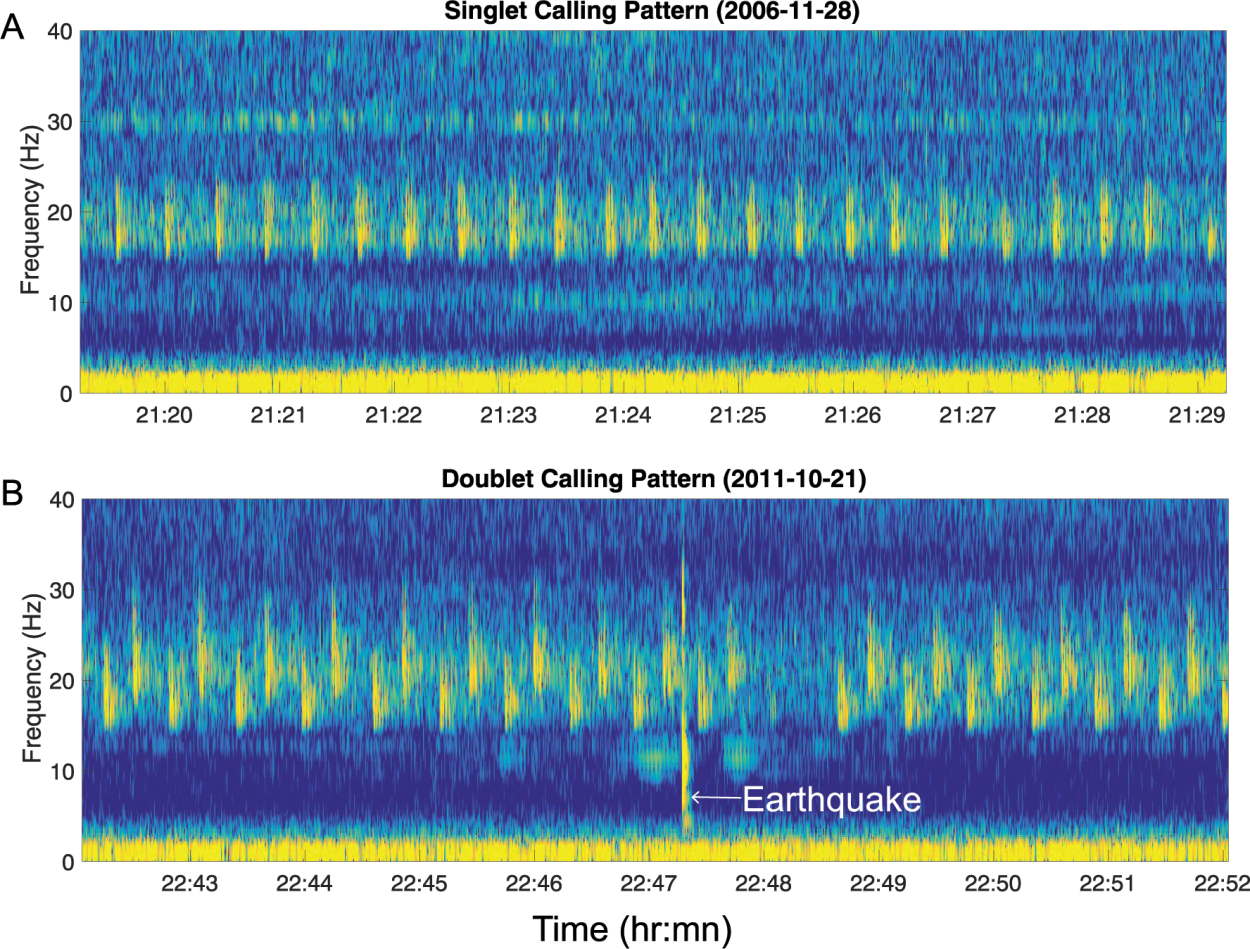
Spectrograms from the Axial instrument showing examples of the two primary song sequences observed. (A) Sequence with one primary frequency and a single IPI (singlet song). (B) Sequence with alternating notes, with two frequencies and two dominant IPIs (doublet song). There is also an earthquake in this record.

Prior work on fin whale signals in the northeast Pacific [18,41,49,50], and our visual inspection of samples of the data, found three patterns (Fig 2): repeated sequences with a single IPI (singlet song), repeated sequences with two alternating IPIs (doublet song), and irregular sequences with variable IPIs. The singlet and doublet sequences have notes with frequencies in the 15–35 Hz band with the highest-amplitude portion of all notes below 30 Hz. Irregular sequences have been attributed to groups of transiting whales [18,50] and can include calls that extend up to about 40 Hz. Since our goal was the analysis of fin whale song, we optimized our detection for singlets and doublets.

**Fig 3:**
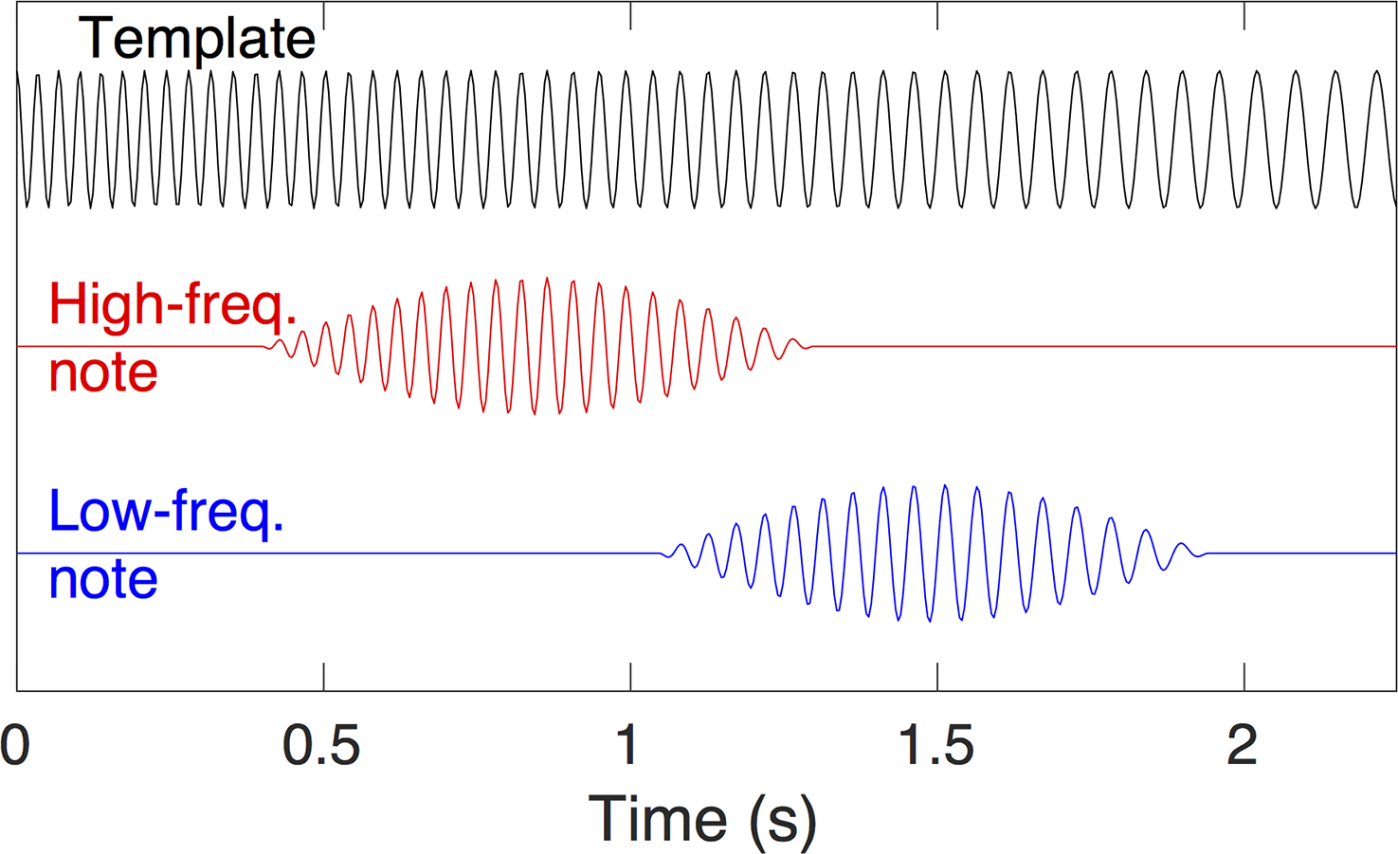
Call template used for detection with a matched filter and examples of two synthetic notes aligned with the filter. The template lasts 2.3 seconds and matches the full range of frequencies seen in all notes. Notes with different frequencies will align differently with the template so after detecting a note with the matched filter, its time is set to that of the maximum amplitude of the note.

Fin whale calls were automatically detected in the time domain using a matched filter [51]. The template note (Fig 3) consisted of a linear chirp decreasing from 30 to 15 Hz over a duration of 2.25 s and was designed to span the observed range of frequencies of the high-amplitude portion of fin whales notes in song sequences and to match the observed rate at which notes sweep down in frequency. Prior to cross-correlating the records with the template, a 14–35 Hz bandpass filter was applied to records with a sample rate of ≥100 Hz and a 14 Hz high-pass filter to records with a sample rate of 50 Hz.

Acoustic data were cross-correlated with the template in 30-min segments and a noise level for the cross-correlation was set to its 90th percentile. All peaks in the cross-correlation that were at least 12 dB above the noise level (i.e., a signal to noise ratio of ≥4) were selected to create a preliminary set of detections. The time at which the template aligned with a note in the cross-correlation is dependent on the note frequency (Fig 3); low frequency notes will align earlier in the cross-correlation. To remove this frequency dependent bias, we computed the envelope function of the filtered record using the Hilbert Transform [52] and then adjusted detection times by up to ±0.5 s so that they matched a peak in the envelope function.

Earthquakes often triggered the detector but were recognizable because they had energy in frequencies below the fin whale band. To eliminate earthquakes, we examined the power spectrum of a 1-s window centered on each detection and eliminated a detection if the average spectral power was higher in the 7–14 Hz band than the 14–35 Hz band. The detector also often picked multipath reflections of a note that resulted from water column reverberations. To remove these, we required that all detections were at least 5 s apart for the shallowest instrument on Axial Seamount and 10 s apart for other stations. We did this by first selecting the highest amplitude detection in a segment and then removing all detections that occurred at less than the minimum allowed time spacing of the selected detection. We then iteratively selected the next highest amplitude detection and removed any detections too close to it, until all detections were either selected or removed. This yielded our final dataset of fin whale note times.

To obtain note frequencies, a spectrogram was computed for each 30-minute segment of the input time series (the FFT used a 1-s Hann window with 90% overlap). The note frequency *F*_*n*_ was estimated for each note using the weighted mean of a subset of the spectrogram within ± 0.5 seconds of the call detection time, and within 15–35 Hz bandwidth of notes in song sequences. This estimate can be expressed analytically as:

$$F_{n}=\frac{\int_{T-0.5}^{T+0.5}\int_{15}^{35}A(f,t)f\;df\;dt}{\int_{T-0.5}^{T+0.5}\int_{15}^{35}A(f,t)\;df\;dt}$$

where *T* is the note time in seconds and *A* is the spectrogram as a function of frequency *f* and time *t* in seconds and Hertz, respectively.

For notes measured from the Cascadia Initiative instruments, the 50 Hz sampling rate limited the upper frequency of observations to 23.5 Hz (the cutoff frequency of the anti-alias filter). Higher frequency notes were detected because they contain significant energy below 23.5 Hz, but the frequency estimates are skewed too low since the full note bandwidth was not used in the weighted frequency estimation. We therefore did not analyze note frequency for these instruments.

### Computing note statistics

**Fig 4:**
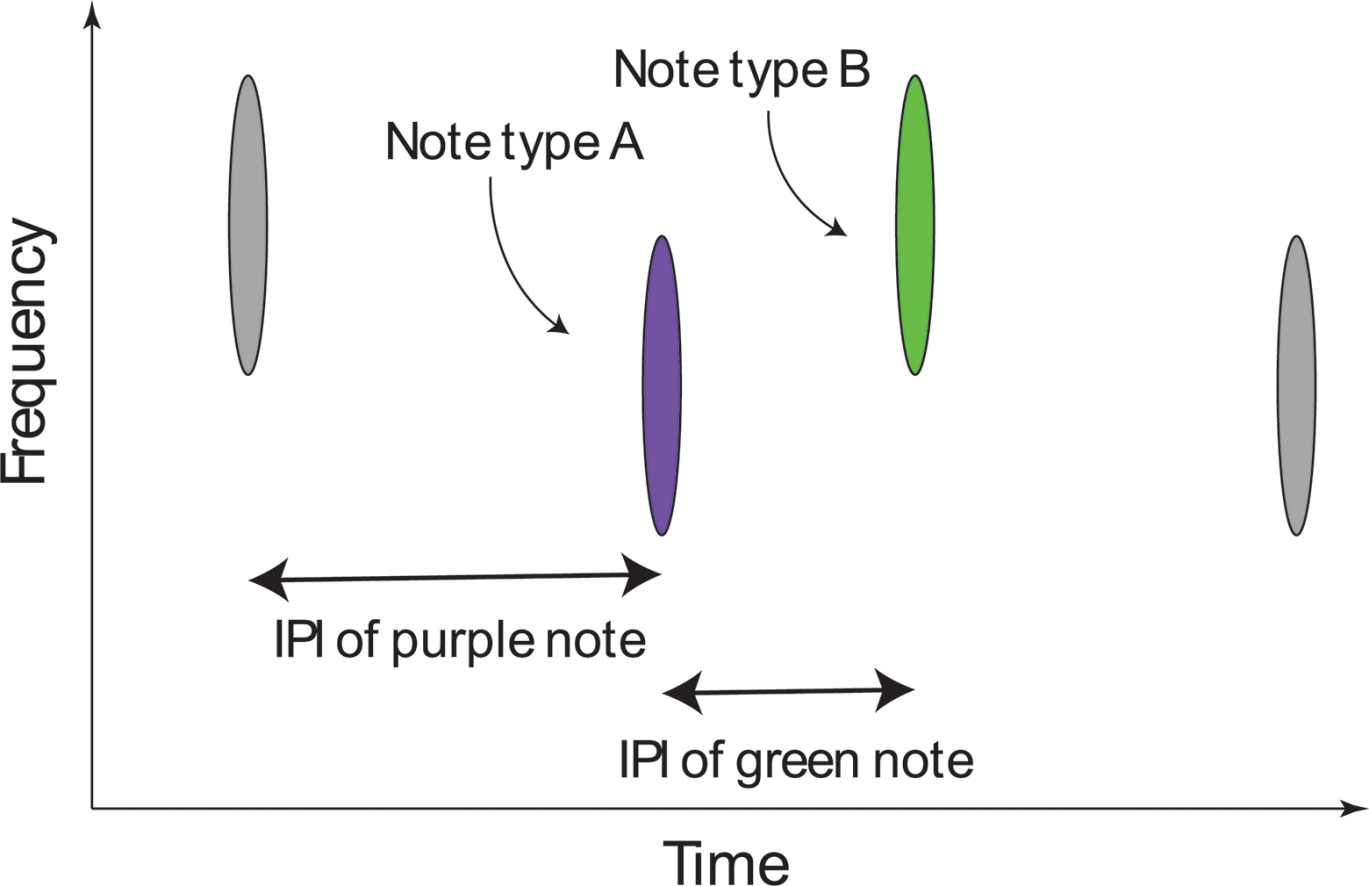
Illustration showing the relationship between calls and IPI. Axes indicate frequency and time, so this schematic is analogous to a spectrogram representation. The green, purple and gray symbols indicate fin whale notes. A-type notes have a lower frequency than B-type notes. The IPI of a given note is the time between that note and the immediately preceding note, irrespective of note type.

We calculated the IPI as the time difference between successive detections and assigned the IPI to the note at the end of the IPI (Fig 4). We discarded all IPIs >40 s and kept only those IPIs that were part of a sequence of at least 20 IPIs with no gaps in singing longer than 20 minutes.

To visualize the relationship between frequency and IPI, we computed a two-dimensional histogram for each month in each singing season (i.e., November–March) and for each full singing season. IPI was binned from 5 s to 40 s in increments of 1 s. Frequency was binned from 15 Hz to 26 Hz in increments of 0.4 Hz. The focus of our analysis was on overall song patterns rather than on absolute number of notes in a given month. To normalize for the large degree of variation in total notes among different months, we scaled note counts to reflect the proportion of notes, as a percentage, relative to the total detections within that month. Peaks in the two-dimensional histogram indicate distinct note-type patterns in frequency-IPI space. To allow for further quantitative analysis, we extracted the coordinates and corresponding counts for each peak by finding local maxima using SciPy’s image processing maximum filter tool [53]. If a peak had an amplitude normalized to the number of calls in the histogram that exceeded 2% per 1-s 1-Hz area of the frequency-IPI plot, the peak was retained for further analysis (if one peak of the doublet sequence met this threshold both peaks were retained).

### Verification

Our method and the controlling parameters for note detection were selected based on a careful inspection of many notes and song sequences, but it is important to evaluate how well our automated method worked. To do this we selected a subset of 50 songs from instruments with sample rates ≥100 Hz that were distributed throughout our 10-year dataset and throughout the singing season. Each selected song lasted at least an hour and we analyzed just the first hour. We compared the automatic detections with manual detections made from spectrograms of unfiltered data, and identified both false and missed detections. We then filtered the records for the 50 songs with a 14–23.5 Hz bandpass filter to simulate the upper frequency content of Cascadia Initiative data, repeated the automated detection analysis, and determined how many additional detections were missed.

The results of the method verification are presented in Table 2. For singlet and doublet songs, about 4–5% of the notes were missed by the automatic detector. This is a result of songs in which some lower amplitude notes fell below the 12-dB detection threshold. The percentage of missed detections can be reduced substantially by selecting only those songs in which the average detection amplitude is well above 12 dB but we chose not to do this because this reduced the size of the IPI dataset and the missed detections did not affect our results for the frequency and IPI characteristics of fin whale songs. We found no examples of notes in regular sequences that were missed because they fell outside the bandwidth of the detector or that were misclassified as earthquakes. Overall, the false detection rate was about 1.4% and was the result of both earthquakes with substantial high frequency energy, and unidentified noise events. The earthquake and fin whale discriminator could be improved by adjusting the threshold for the spectral test and looking at parameters such as spectral bandwidth but we chose not to do this because randomly located false detections did not impact our results. Our verification showed that the use of the CI data with a 50-Hz sample rate does not significantly affect the detection of calls in singlet and doublet songs.

**Table 2:** Results of the method verification.

	Sequence Type				All Sequences	
	Singlet Notes		Doublet Notes			
**Manual**						
Detections	1335		1404		2739	
						
**Automated**						
Detections	1290		1357		2647	
Missed	59	(4.4%)	72	(5.1%)	131	(4.8%)
False	14	(0.4%)	25	(1.8%)	39	(1.5%)
Earthquake	10		23		33	
Noise	4		2		6	
						
**Automated 23.5 Hz Lowpass Filter**						
Additional Missed	0	(0.0%)	3	(0.2%)	3	(0.1%)

The table shows the number of note detections in different categories for manual and automated analysis of 50 1-hour song sequences distributed throughout the 10 years of data. Percentages of missed automated calls are relative to the number of manual detections and percentages of false detections are relative to the number of automated detections.

We also compared the times of detections obtained with the automated methods for 14–35 Hz and 14–23.5 Hz bandpass filters and a manual identification of peak amplitude on a spectrogram. We looked at over 100 calls distributed through our test dataset and found the times were always consistent within 0.1 s.

## Results

### Song and note types

Over 1.8 million notes from more than 8,000 song sequences recorded at 8 sites in the northeast Pacific Ocean were used to examine singing patterns in fin whales from 2003–2013. Two patterns were recorded most frequently at all instruments and can be best described as consisting of combinations of two distinct notes. For simplicity, the two pattern types are hereafter referred to as singlet (IPI > 22 s) and doublet songs (IPI < = 22 s), and the note types are referred to as A (< 22 Hz) and B (> = 22 Hz). Examples of the two song types are shown in Fig 2A and 2B. Singlet songs consisted of a single, repeated A note with a fixed IPI and frequency (Fig 2A). Doublet songs had two primary notes, alternating between a low frequency, high IPI, A note, and a higher frequency, low IPI, B note (Fig 2B).

Singlet songs were more common in earlier years of the study, with a gradual transition to doublet songs over the decade of recording. From 2003–2009, although singlet songs were more common, doublet songs also occurred in the same months and years. The introduction of the higher-frequency notes would effectively reduce the IPIs of the original sequence. In this transitory period, note type A remained consistent while being “interrupted” by note type B. Since note type B did not interrupt at exactly halfway between two A-notes, it resulted in a bi-modal distribution of IPIs.

### Decadal trends

#### Axial

**Fig 5:**
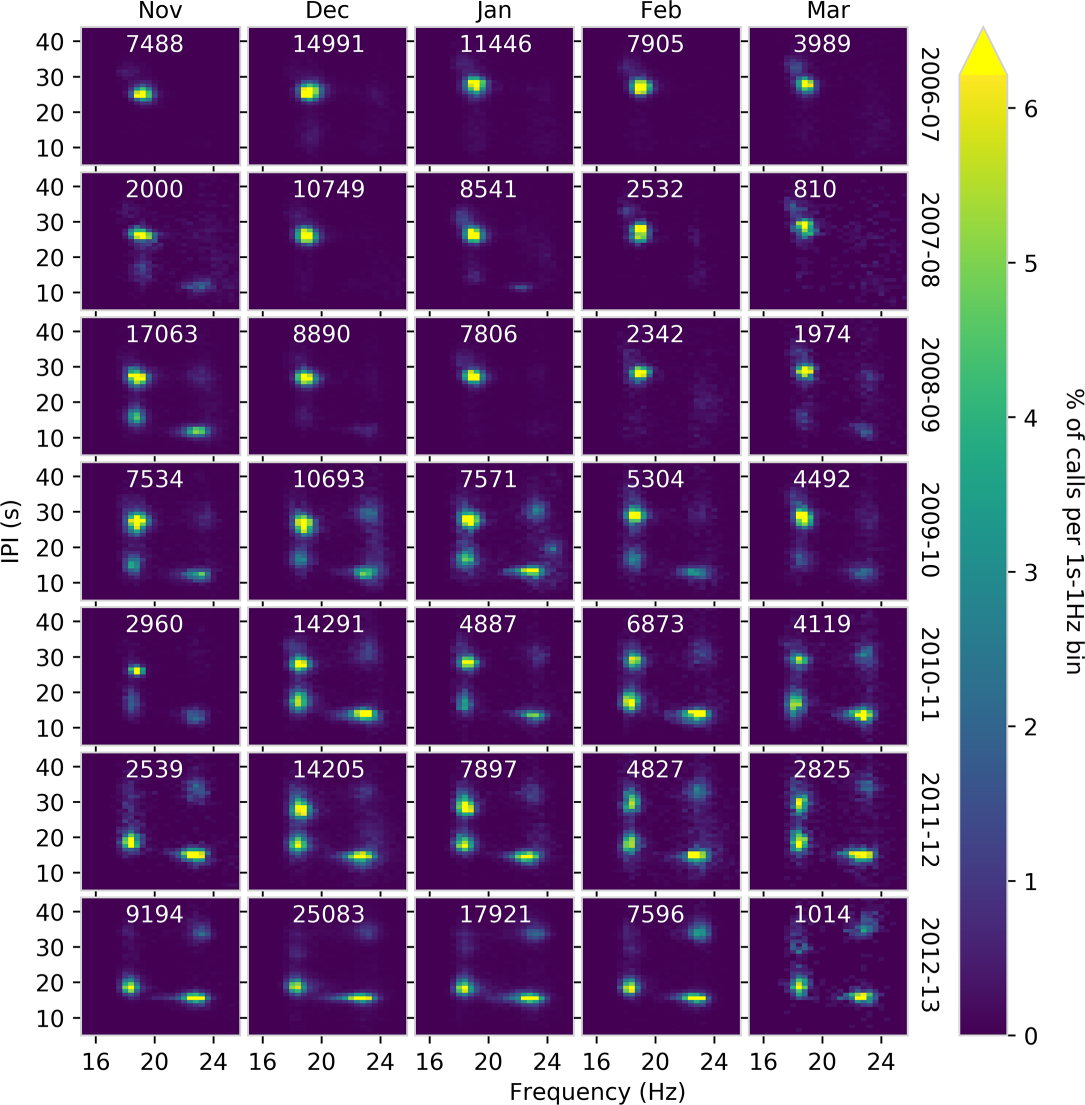
Monthly two-dimensional histograms of IPI and frequency for the Axial OBH from 2006–2013. Frequency is plotted along the x-axis and inter-pulse interval (IPI) along the y-axis. Colors correspond to density of calls in 1-s 1-Hz areas of the plot as a percentage of the total calls in the month (white labels).

**Fig 6:**
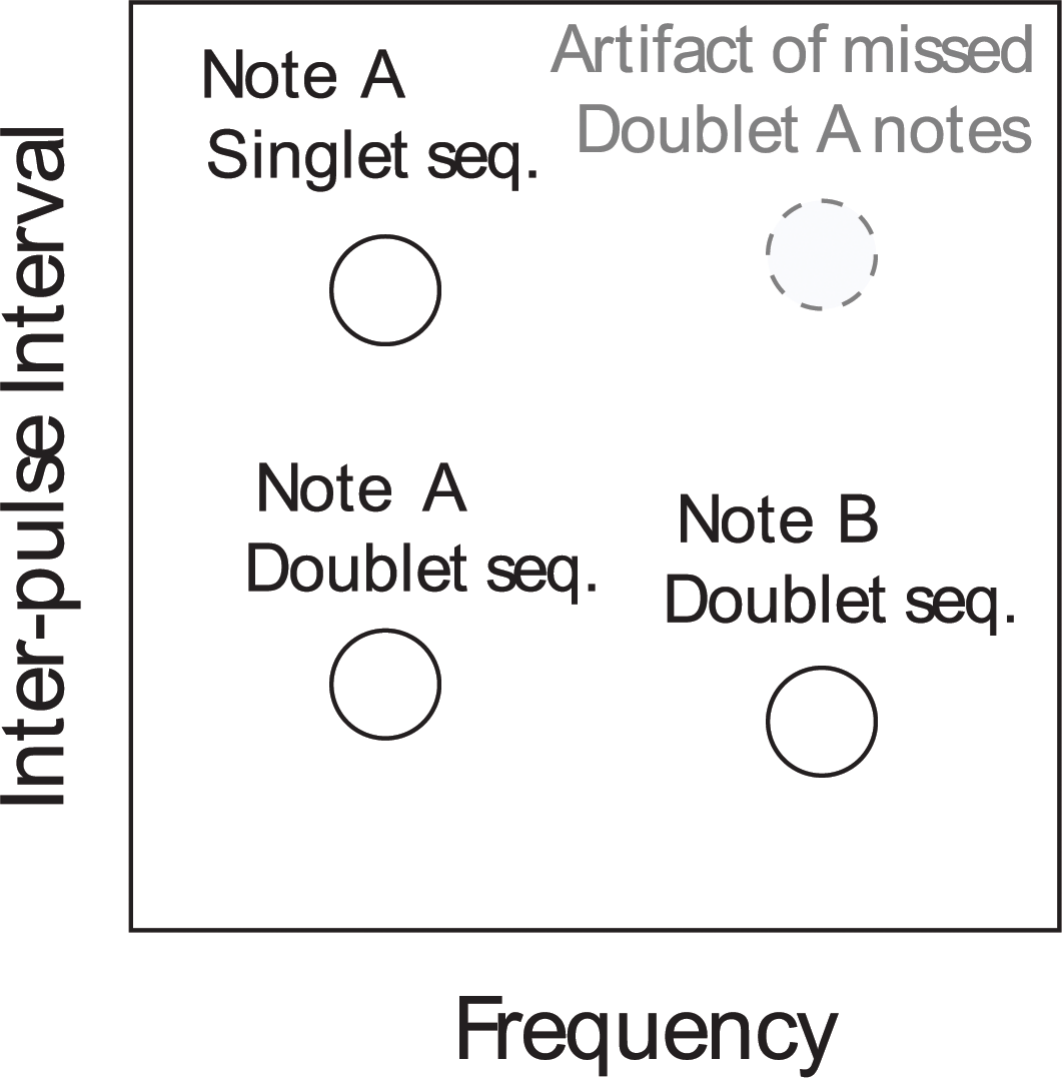
Illustration showing the relationship between peaks in two-dimensional frequency-IPI histograms and the singlet and doublet songs. Following Fig 5, frequency is plotted along the x-axis and inter-pulse interval along the y-axis. A-note singlet songs, with higher IPI and lower frequency appear in the upper left. A notes from doublet songs have a similar frequency but appear in the lower left. B notes from doublet songs appear in the lower right. The peak that is sometimes observed in the upper right is an artifact and it occurs when lower amplitude A notes that are part of A/B doublet songs are not detected.

At the Axial site, there was a clear inter-annual transition from singlet songs to doublet songs (Fig 5). The two-dimensional histograms for each month and year at the Axial site illustrate the predominance of singlet songs in 2006–2009 and the change over time to doublet songs (Fig 5). Fig 6 provides an illustration to demonstrate how the frequency and IPI viewed on the two-dimensional histograms can be interpreted and related back to the singlet and doublet song sequences shown in Fig 2A and 2B, respectively. In earlier years (2006–2009), the dominant note had an IPI of 25–30 seconds and frequency around 18 Hz (Fig 5). This can be observed in the histograms as a single bright peak (e.g., January 2007) that represents note A of a singlet song (Fig 6). Gradually two additional peaks appear in the histogram with IPIs between 10 and 15 s, one with a frequency of ~18 Hz and one with a frequency of ~23 Hz (Fig 5). These are the A and B notes of the doublet song (Fig 6). This song was observed early in November 2007 and November 2008 and throughout the season starting in 2009–2010 and progressively became more prominent, although singlet songs continued to be observed at high levels until 2012–2013 when they essentially disappeared Fig 5). In the doublet song, A notes were often lower in amplitude than B notes (Fig 2B) and were occasionally not detected. This resulted in the weak artifact peaks (Fig 6) seen in the upper right quadrant of some months (e.g., March 2011).

#### Additional recorders

**Fig 7:**
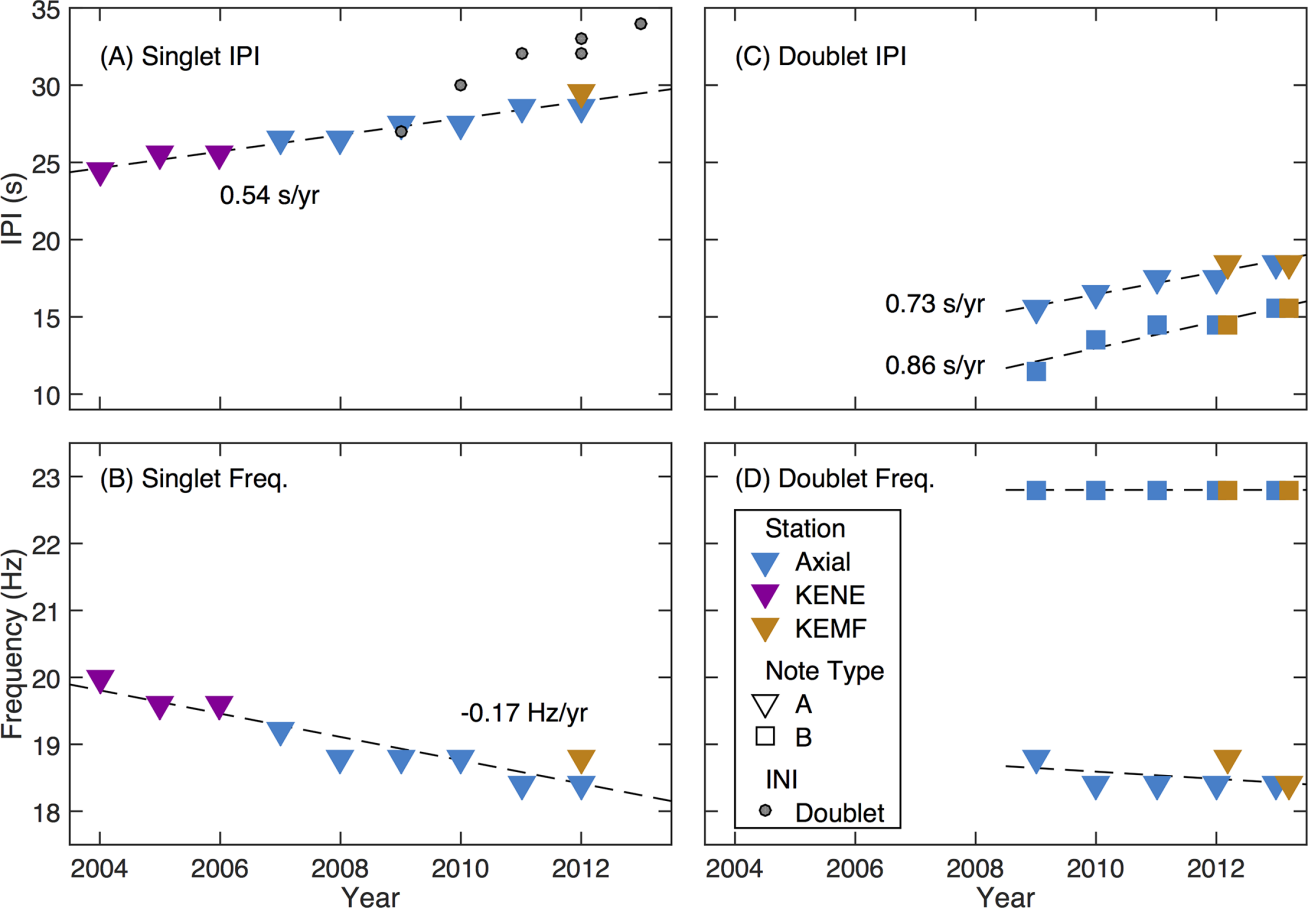
Frequency and IPI characteristics from three instruments plotted as a function of year from 2003–2013. (A) IPI for the singlet song, (B) frequency for the singlet song, (C) IPI for the doublet song, and (D) frequency for the doublet song. Results are colored by station (Axial, blue; KENE, purple; KEMF, orange), and note type is indicated by shape (Note type A, triangles; note type B, squares). Dashed lines show least-squares straight line fits to the data which are labelled with the slope when it is significantly different from zero. In (A) gray circles show the inter-note interval (i.e., sum of the IPI for the two doublet notes). X-axis year labels correspond to the start of the year and results for a calling season (November-March) are plotted at the start of January in that season (i.e., the point at 2010 corresponds to the 2009–2010 calling season).

The Axial dataset covered seven years (2006–2013), and data from nearby KENE (200 km to the NNE of Axial) were available from 2003–2006. Inclusion of these data extended temporal coverage to a full decade. To justify the use of these additional data, we looked at data from KEMF, which was located 14 km from KENE, but had temporal overlap with Axial data (2011–2013). The song data from KEMF showed the same characteristics in both frequency and IPI as the Axial data (Fig 7), thus we believe combining KENE and Axial for a full decadal analysis was justified.

Both monthly IPI and frequency changed over time from 2003–2013 (Fig 7). Only singlet songs consisting of A notes were recorded at the start of the decade. The introduction of note B and doublet songs began at very low levels as early as 2005–2006, became well-established early in 2008–2009, and became prominent from that point onward. The IPI of A-note singlet songs increased significantly, at a rate of 0.54 s/year (the coefficient of determination for the straight-line fit, R^2^ = 0.96; and the probability that the slope is not significantly different from zero as determined by a t statistic, p < 0.001) over the decade of observation (Fig 7A). The frequency of the same subset of sequences decreased significantly by 0.17 Hz/year (R^2^ = 0.86, p = 1.1 x 10^−4^) over the decade (Fig 7B). We also looked at the trends in doublet songs, although restricted our analysis to 2007–2008 and onwards, since that was when the doublet songs occurred most consistently. Within that subset, we observed statistically significant increases in IPI for both the doublet A and B notes. IPI of doublet A notes increased at a rate of 0.73 s/year (R^2^ = 0.91, p = 0.001) and doublet B notes increased at a rate of 0.86 s/year (R^2^ = 0.90, p = 0.001). Frequencies of the doublet song notes did not show any significant change over the same time period. When doublet songs first appear as a prominent feature in 2008–2009, the sum of the inter-note interval for doublet matches the IPI for the singlet (Fig 7A). This suggests that the doublet result from consistent interruptions of the singlet song IPIs. However, in later years the inter-note interval for the doublet song exceeds that of the singlet song (Fig 7A).

To get a sense of the uncertainties in the frequency and IPI associated with each data point in Fig 7, we measured the widths of the peaks in the frequency-IPI histogram in the frequency and IPI direction, respectively, at half of their maximum amplitude. We assumed that the underlying distributions were Gaussian, so the full width at half amplitude can be divided by 2.36 to obtain an estimate of the standard deviation of the data. This yields standard deviations for IPI between 0.7 s and 2.5 s and for frequency between 0.4 and 0.7 Hz. Since each histogram is based on several hundred sequences, we infer that the standard error for each data point is much smaller, and thus that the uncertainty in IPI and frequency is half the discretization interval in the histogram (i.e., 0.5 s for IPI and 0.2 Hz for frequency).

### Seasonal and inter-annual trends

**Fig 8:**
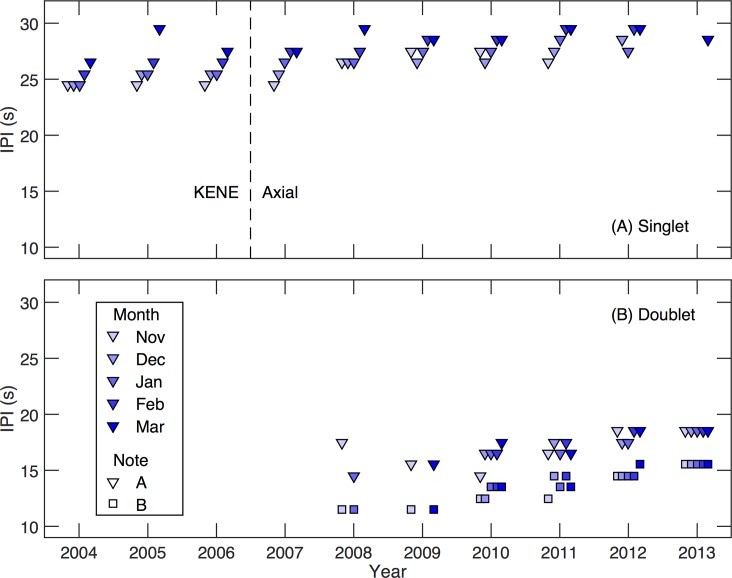
Monthly IPI peaks from KENE and Axial. Results for (A) Singlet songs and (B) doublet songs illustrate the variations in IPI within years and from year to year. Colors indicate month from November to March, A notes are indicated by triangles, and B notes are indicated by squares.

In addition to the decadal trends described in the previous section, within-year patterns in IPI were also observed. Between 2003–2004 and 2010–2011, there was a within-year increase in IPI by month, followed by a reset to a shorter IPI (that was nevertheless greater than that of the same month the year prior) at the start of the following calling season (Fig 8A). The pattern became less clear as doublet song types first appeared in 2007–2008, and by 2011–2012, the monthly increase in IPI was no longer clearly observed. In contrast to the singlet songs, there is no clear seasonal change in the IPI for the doublet song (Fig 8B).

### Geographic trends

The decadal trend in IPI and frequency of notes in both singlet and doublet songs is clear at Axial and the Endeavour segment locations (KENE/KEMF). Because we had access to data from locations to the east and south of the Axial/Endeavour region, but from fewer years, we wanted to examine if the annual patterns in song type occurrence and IPI were robust over a greater geographic range by including analysis from CI and COLZA experiment sites (Fig 1). As the CI OBSs only had an upper limit on bandwidth of ~23.5 Hz, and our estimates of frequency could be biased low (as described in the methods), we chose to focus only on the IPI values for the geographic analysis of the CI data. Other studies that have examined small-scale geographic variation in fin whales have relied on IPI alone to establish population identity ([16,35], but see [38], and [36] for the use of frequency components for geographic distinction).

**Fig 9:**
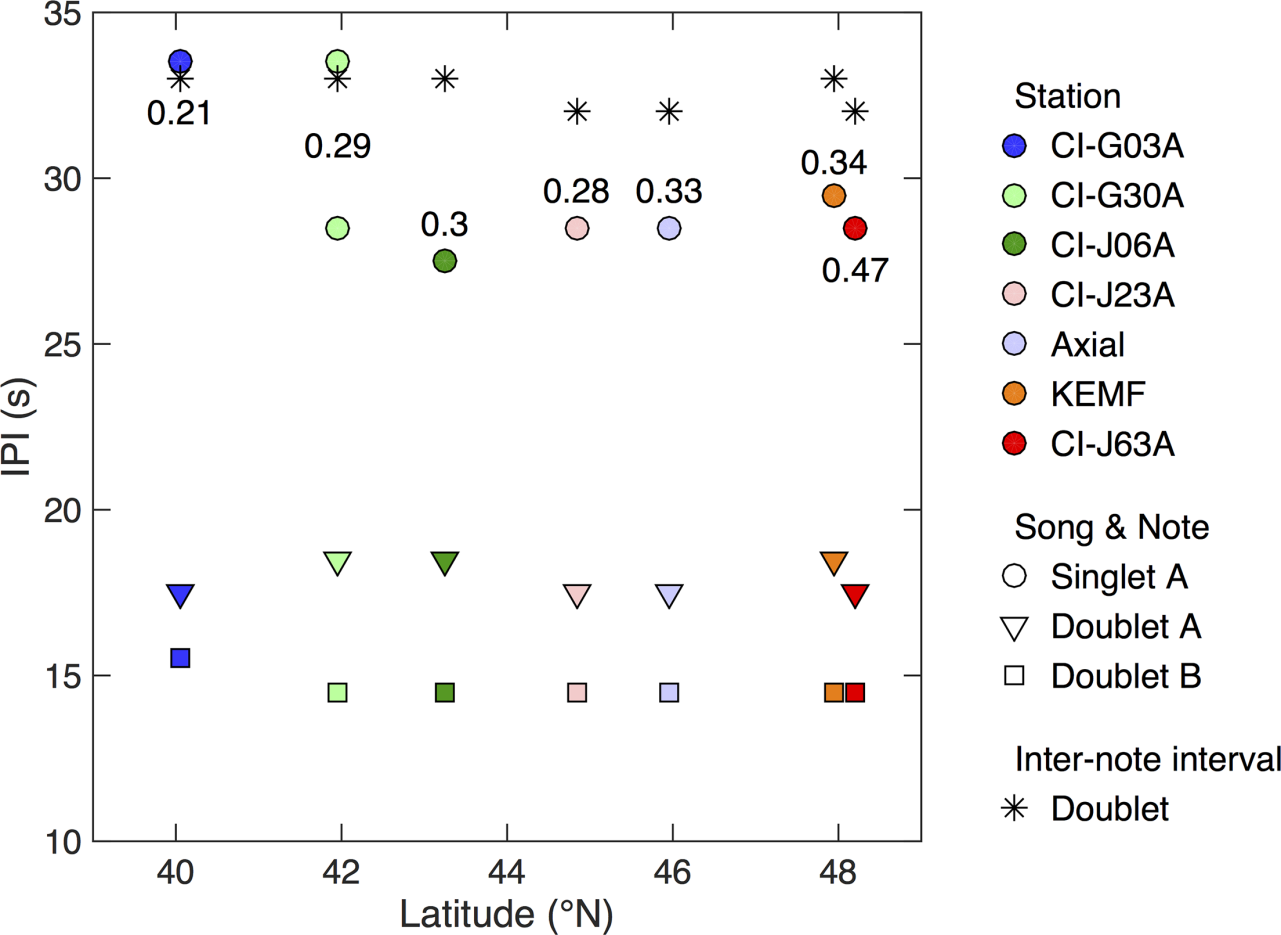
Annual IPIs as a function of latitude recording during the 2011/2012 calling season. Singlet A-notes are indicated by circles, doublet A-notes are indicated by triangles, doublet B-notes are indicated by squares, and the doublet inter-note interval by asterisks. Colors correspond to station and labels indicate the fraction of notes that belong to singlet sequences.

IPIs for each location were extracted from two-dimensional histograms and were computed over the entire 2011–2012 calling season for singlet A notes, doublet A notes, and doublet B notes (Fig 9). Although we did not observe a significant geographic trend in the IPI of doublet songs, there appeared to be two distinct groupings of singlet A song IPIs. At the southern two stations (latitudes ≤42°N) a singlet song with an IPI of 33 s was observed. At all but the most southern station (latitudes ≥42°N), a singlet song with an IPI of 28 s was observed. Both singlet songs are present at CI station G30A. A two-tailed t-test showed a statistically significant difference in the IPI between the higher and lower IPI singlet songs (p<0.001). There was also a north to south decrease in the proportion of singlet songs (Fig 9) with about twice the proportion of singlet calls at the northern two stations than the southern station.

**Fig 10:**
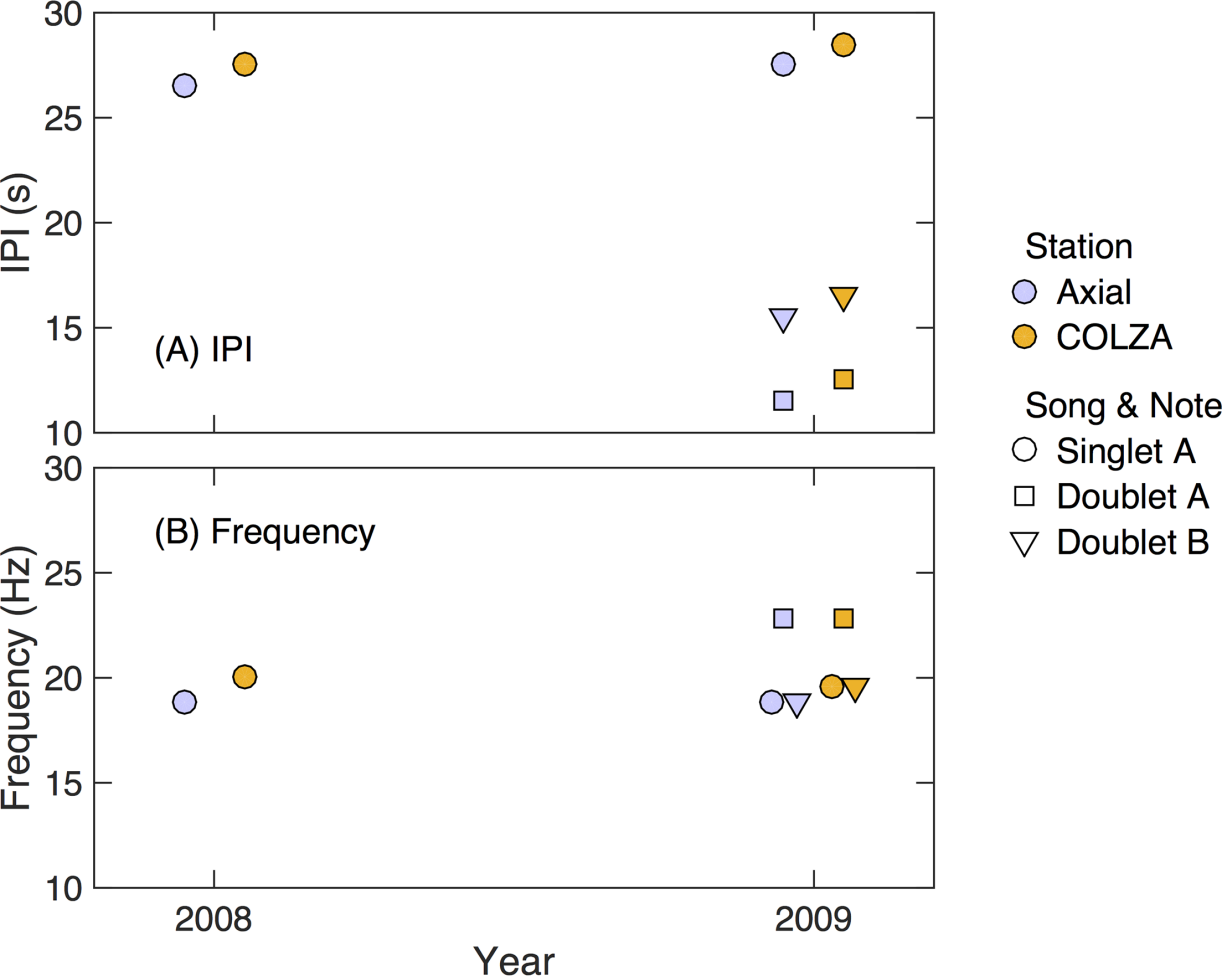
Annual means of frequency and IPI values at the COLZA and Axial sites for the 2007–2008 and 2008–2009 calling seasons. Station is indicated by color. Doublet A-notes are indicated by triangles, doublet B-notes are indicated by squares, and singlet A-notes are indicated by circles. Symbols have been offset horizontally from one another so that they all display.

A similar analysis was carried out for the 2007–2008 and 2008–2009 data for the Axial and COLZA instruments. The COLZA instrument was located approximately 350 km to the east of Axial, just west of the foot of the continental slope (Fig 1). Both the COLZA and Axial instruments sampled data at rates that allowed for the analysis of both frequency and IPI. Singlet songs dominated both Axial and COLZA instruments, although some doublets were observed in both years (Fig 10). During the 2007–2008 calling season, fewer than 5% of songs were doublets, and these smaller peaks in the two-dimensional monthly histograms were not reliably detected by the automatic algorithm, so are not included in the analysis. By 2008–2009, doublet songs made up ~24% of all recorded songs at COLZA, and ~16% of all recorded songs at Axial. Both the IPI and frequency of the singlet and doublet calls are statistically indistinguishable from one another at this site.

## Discussion

It is clear, from this work and other recent work on fin whale acoustics [41,43,37], that fin whale sound production is more complicated than previously thought. Our work adds further support for intra-annual changes observed in inter-pulse interval [41]. In the northeast Pacific, IPI increased over the course of each month from November through March, but then partially reset to the previous year’s value at the start of the following year. Over a decade however, the IPI for all song types increased significantly inter-annually such that from the 2003–2004 to 2011–2012 calling seasons, the IPI of singlet songs increased from 24.5 to 29.0 s. Over the same time period, the peak frequency decreased significantly from 19.8 Hz to 18.3 Hz. Further, over the ten-year span from the 2003–2004 to 2012–2013 calling season, an additional note type was introduced and became more prominent. The two note types then alternated in a doublet pattern resulting in a distinctly different song. By the 2012–2013 calling season, the doublet song was the only song present at significant levels.

A similar pattern of an annual change in frequency with a partial reset and overall decadal decrease in frequency has been documented in Antarctic blue whales recorded off Australia [34,54]. A decrease in frequency in numerous blue whale populations over time has also been noted. McDonald et al. [33] explored a number of potential factors to explain the observed decline in tonal frequencies, and hypothesized that increasing population size or increases in ambient noise were possible explanations for decreasing frequencies. Garilov et al. [54] suggested that the underlying explanation for the shift is likely more complex than an increase in animal density and hypothesized that the inter-annual shifts might be related to changes in calling depth. Other marine mammal species have shown evidence of long term shifts in call frequency that have been attributed to anthropogenic noise, although these tend to be associated with an increase in frequency, rather than a decrease such as we observed. For example, Parks et al. [55,56] observed a long-term increase in right whale calling frequency under high noise conditions. Foote et al. [57] describe a terrestrial analog, whereby birds were observed to sing at higher pitch in the presence of urban noise. To date, decreases in call frequency have not typically been attributed to increasing environmental noise. Further, recent long-term studies of ambient noise in the northeast Pacific Ocean have documented both increases and decreases in ambient noise due to shipping over the time period covered by this study [33,58,59].

A recent study of fin whale calls recorded between 2000 and 2006 found within-year increases in IPI from October to February in three regions: southern California, Hawaii, and the southeast Bering Sea [41]. Like our study, at least for the southern California region, there was an annual reset from the maximum IPI in February (in our dataset, March) to a minimum in October. That study had 3 consecutive years of data from southern California but did not find any inter-annual trends. The data from Hawaii are intriguing in that for the month of December the median IPI increased from 2000 to 2005 (although only the December median IPIs are reported for the later calling season). The Bering Sea location, however, did not show a corresponding increase over the same time period. Koot [49] also described a within-year increase in IPI for doublet song of approximately 2.6 s from August to January for fin whales off British Columbia in 2010–2011.

Oleson et al. [41] hypothesized that the within-year IPI trends might be linked to reproductive hormonal activity, and posited that a shorter IPI can be considered equivalent to a greater calling rate and therefore evidence for increased male fitness either as a mate attractant or to mediate male-male assessment. Either (or both) of these motivators could result in a lower call rate later in the season and the argument presented by those authors is intriguing and well supported by examples from other taxa. It does not help explain, however, the decrease in frequency and overall increase in IPI or the emergence of a second song type in our data. In doublet songs, the IPIs were necessarily decreased and did not change seasonally from the 2010–2011 calling season onwards. It may be that there is a fitness tradeoff between the center frequency of a signal and its repetition rate. For instance, if it is energetically more expensive for fin whales to call at a lower frequency, animals might call less often, thus increasing their IPI. Conversely, calling more often might require producing sounds at a higher frequency. Without further knowledge of the behavioral ecology of fin whales, the hypothesis attributing seasonal changes in note characteristics to reproductive activity cannot entirely explain the longer-term trends that we observed. These trends over longer time scales might reflect acoustic behaviors that are spread geographically throughout the region.

Thompson et al. [20] were the first to aggregate 20-Hz pulse characteristics from around the globe to highlight the potential for geographic variation in fin whale calls. Hatch [60] examined acoustic and genetic data from fin whales from several sites in the Pacific and Atlantic Oceans, and found significant regional acoustical differences in call characteristics. Our timeline overlaps with that of the Oleson [41] study during 2005–2006, although the instruments they used were far from our study (the Bering Sea and off Hawaii). The IPIs they reported during that time period were between ~25–35 s which is not substantially different from our results from the Axial dataset over the same time period (24.5–30.5 s).

Our study illustrates that if song characteristics are going to be used for determining population structure in fin whales, then long term, decadal-scale data need to be considered, or lacking this, data from the same months and the same year should be compared to avoid potentially identifying populations as acoustically distinct when they are not [41]. Additionally, instrument locations need to be sufficiently dense in order to capture geographic variations and difference between these and temporal changes, which occur simultaneously and could otherwise be difficult to distinguish. For instance, the median “long” IPIs from December 2000 and February 2001 from southern California, Hawaii and the Bering Sea were all different [41]; whether these differences are robust enough to delineate populations will require more data.

The results from our geographic analysis of the 2011–2012 data show that there was a significantly lower singlet song IPI at the northern stations than at the southern stations in the network although doublet song IPI remained the same across the same boundary. The two middle latitude locations (CI-J06A and CI-J23A) show both types of singlet IPI (short and long). Off the northwest coast of Vancouver Island [49], both singlet and doublet songs were observed in 2010–2011, with average doublet IPIs of 12.9 s and 17.3s, which is consistent with IPIs observed in our study. IPIs between consecutive lower frequency notes (which we refer to as A notes) was 28.8 s [49]. Although the study location was at a slightly higher latitude (~50°N), and in coastal waters (~105 m water depth), the singlet song IPI was similar to the singlet A song IPIs observed in our higher latitude data. The distinct shift in singlet A note IPI is interesting since it reveals a difference in song characteristics between the northern and southern portions of our experiment region, which may be evidence of two acoustically distinct groups of fin whales [37].

The 2007–2009 comparison between the COLZA and Axial sites allowed us to explore frequency and IPI at locations with similar latitude, but differences in proximity to the coast. The frequency and IPI are statistically indistinguishable between the two sites consistent with a single acoustic population. It would be informative to look at frequency characteristics of both singlet and doublet calls across a broader geographic area and over multiple years to determine how consistent the calling characteristics are both spatially and temporally.

The two types of shifts in our dataset–a gradual shift in IPI and frequency, and a more distinct shift from one calling pattern to another–could hypothetically be a result of cultural transmission. Boyd and Richardson [61] define culture as “information or behavior acquired from conspecifics through some form of social learning”. One way of studying animal culture is by observing behavioral change patterns in wild populations where the animals’ genetics or environment are not clear causes [62]. Cultural transmission has been observed in cetaceans [27,29,63,64], but also in terrestrial animals such as frogs [65], bats [66], and birds [67–69].

It is difficult to determine the causes of the decadal-scale variations observed here because we have a very limited understanding of the underlying population structure of fin whales. We can only speculate based on what we know about fin whale distributions and migratory movements, as well as what has been observed in other species, both marine and terrestrial. Payne and Webb [70] suggested that fin whales might maintain acoustic contact over great distances and this ability allows them to be unconstrained by geography with regards to potential breeding aggregations. In this way, whales from different North Pacific populations could potentially interact on regions of high secondary productivity and possibly exchange or, as suggested by Oleson [41], synchronized song types. Several populations of marine mammals have been observed to adopt a new song type after encountering even a small number of ‘foreign’ singers. For example, Noad et al. [29] describe humpback whales off the Australian east coast gradually adopting song originating from humpback whales off the Australian west coast over a period of three years. Over an 11-year observation time, several humpback whale song types in the western and central South Pacific were observed to transmit uni-directionally across multiple populations from east to west [27]. Stafford and Moore [71] observed a blue whale in the Gulf of Alaska that produced a song that combined features of both the eastern and western north Pacific blue whale populations, suggesting that blue whales can mimic each other’s calls. Resident killer whales observed off the coast of British Columbia showed evidence of a gradual shift in one of the two call types studied over a duration of 13 years [63]. The authors hypothesized that it might be evidence of cultural drift in the call structure, combined with transmission of the new call characteristics between members of the population.

In order to test the hypothesis that fin whale vocalizations are undergoing cultural transmission, it would be necessary to continue to simultaneously explore more data from sufficiently dense networks covering wide regions over long time periods. Mizroch et al. [72] suggest that there are two subpopulations of fin whales in the North Pacific Ocean: one western and one eastern. At least a subset of these are believed to mingle on the northern summertime feeding grounds, which would potentially allow them the opportunity to meet and exchange song types. It would be interesting to explore and compare long term calling characteristics from non-migratory populations, such as from the Gulf of California [73], or the East China Sea [74] where we would expect that calling characteristics would evolve separately. Reliably linking acoustic observations to cultural transmission would require a much more thorough understanding of the underlying geographic population structure, behavioral ecology, and migratory patterns of the animals being studied. It would also be informative to look at whether long-term trends in frequency and IPI are observed in other ocean basins around the world.

### Analysis limitations

One of the sources of uncertainty arises from the CI dataset. All of the instruments in that network had a sample rate of 50 Hz, and an effective upper frequency limit at ~23.5 Hz. Although a large portion of even the higher frequency notes were lower than this, the weighted frequency estimation was biased by the lack of signal above that point. Although having access to higher frequencies could change the result slightly, it is not expected to have a significant impact on the final conclusions as shown by our methods verification where we limited the bandwidth of signals that had higher sample rates to determine what might be missed in our analysis.

To express the variability of monthly estimates of IPI and frequency derived from two-dimensional histograms, we used standard error, where standard deviations in the measurements were scaled by the number of sequences in the corresponding month. This was based on the assumption that each sequence was produced by a different animal, and were therefore independent samples. A more conservative estimate of the number of independent variables would be to use the number of days containing sequences, rather than the number of sequences. If multiple sequences occurred during a given day, those sequences might have been produced by a single animal. When this alternate method of estimating the number of independent samples is used, estimates of standard error increase by a factor of approximately two. This does not affect the interpretation of any of the reported results, but is worth considering. The true number of independent samples is likely bracketed by the number of songs in one month, and the number of days with recorded songs.

The definition of IPI used in this analysis was chosen partially based on convention, and also because it was a straightforward computation. It might be beneficial in future work to compute inter-note interval, that is, the time between successive notes of the same frequency. However, exploring patterns in this way allowed us to easily explore the relationship between frequency and IPI without making prior assumptions. Notes of a particular frequency tended to follow a particular IPI, giving rise to the distinct groupings described here.

In this paper we describe broad patterns in both frequency and IPI for fin whale songs over a decade. Previous research based on more temporally and spatially focused datasets has revealed finer nuances in these parameters. From an in-depth analysis of call types observed at the Endeavour Segment location, four dominant note types were described in terms of both frequency and IPI, and these call types were linked to swimming behavior over an entire year [50]. That study used the same data we used from KENE for the 2003–2004 singing season and reported slightly different frequency and IPI patterns. They described three distinct song types, along with a category of mixed or irregular song types that occurred in months outside of this study. The most commonly observed song type had a dominant IPI peak at 24 s (similar to this study) and a smaller secondary peak at 30 s. Our methodology focused on the more dominant note types, and was not sensitive to the subtler shifts described by Soule et al. [50]. Future work might benefit from a more detailed examination of those notes that occur less frequently.

## Conclusions

The use of calling characteristics to define distinct sub-populations for conservation purposes is more complicated for fin whales than previously believed. There is some evidence indicating that in addition to long term shifts in both frequency and IPI, there are also subtle shifts across the region, in particular for the singlet A note songs. Future studies will need to take into account the potential for both spatial and temporal variability when planning surveys and when interpreting the resulting data.

Future work in the exploration of fin whale IPI and frequency patterns would benefit from the analysis of additional stations both spatially and temporally. The Cascadia Initiative Experiment [48] consisted of around 70 instruments deployed from 2011–2015 off the west coast of Canada and the US. The scope of this study focused on a specific set of five of those instruments deployed for the first year of the experiment. Based on our findings, we suggest that further exploration of this valuable open source dataset would help to better understand geographic variations in calling in this region. Additionally, data from the Regional Scale Nodes [75] and Ocean Networks Canada Neptune [76] cabled observatories already provide several years of ongoing recordings that should also be mined to explore long-term trends.

It would also be useful to conduct similar exploration of recordings in different ocean basins. Seismic instruments such as those used in this study have been deployed in networks around the world and the data from many of these are also openly available. From 2001 to 2015 United States Ocean Bottom Seismograph Instrument Pool (OBSIP) has supported over 750 OBS deployments longer than 250 days and this data is openly available from the IRIS (Incorporated Research Institutions for Seismology) Data Management Center. There are also substantial fleets of OBSs operated by Japan and several European countries that could potentially be explored. OBSs deployed for long durations typically have sampling rates of 50 Hz to 100 Hz, but with improvements in technology higher frequencies are becoming increasingly feasible.

## References

[1] Carretta JV, Oleson EM, Weller DW, Lang AR, Forney KA, Baker J, et al. U.S. Pacific Marine Mammal Stock Assessments: 2014. 2015.

[2] Reilly SB, Bannister JL, Best PB, Brown M, Brownell Jr. RL, Butterworth DS, et al. Balaenoptera physalus: The IUCN Red List of Threatened Species 2013: e.T2478A44210520. 2013.

[3] Watkins WA, Daher MA, Reppucci GM, George JE, Martin DL, Dimarzio NA, et al Seasonality and distribution of whale calls in the North Pacific. Oceanography. 2000;13(I):62–7.

[4] Stafford KM, Nieukirk SL, Fox CG. Geographic and seasonal variation of blue whale calls in the North Pacific. J Cetacean Res Manag. 2001;3(1):65–76.

[5] Stafford KM, Nieukirk SL, Fox CG. Low-frequency whale sounds recorded on hydrophones moored in the eastern tropical Pacific. J Acoust Soc Am. 1999;106(6):3687 10615707 10.1121/1.428220

[6] Mellinger DK, Clark CW. Blue whale (Balaenoptera musculus) sounds from the North Atlantic. J Acoust Soc Am. 2003;114(2):1108–19. 12942988 10.1121/1.1593066

[7] Stafford KM. Two types of blue whale calls recorded in the Gulf of Alaska. Mar Mammal Sci. 2003 Oct;19(4):682–93.

[8] Stafford KM, Nieukirk SL, Fox CG. An acoustic link between blue whales in the Eastern Tropical Pacific and the Northeast Pacific. Mar Mammal Sci. 1999 10;15(4):1258–68.

[9] Nieukirk SL, Stafford KM, Mellinger DK, Dziak RP, Fox CG. Low-frequency whale and seismic airgun sounds recorded in the mid-Atlantic Ocean. J Acoust Soc Am. 2004;115(4):1832 15101661 10.1121/1.1675816

[10] Stafford KM, Chapp E, Bohnenstiel DR, Tolstoy M. Seasonal detection of three types of “pygmy” blue whale calls in the Indian Ocean. Mar Mammal Sci. 2011 10;27(4):828–40.

[11] McDonald MA, Hildebrand JA, Wiggins SM. Increases in deep ocean ambient noise in the Northeast Pacific west of San Nicolas Island, California. J Acoust Soc Am. 2006;120(2):711–8. 16938959 10.1121/1.2216565

[12] Buchan SJ, Hucke-Gaete R, Rendell L, Stafford KM. A new song recorded from blue whales in the Corcovado Gulf, Southern Chile, and an acoustic link to the Eastern Tropical Pacific. Endanger Species Res. 2014 3;23(3):241–52.

[13] Watkins WA, Tyack P, Moore KE, Bird JE. The 20-Hz signals of finback whales (Balaenoptera physalus). J Acoust Soc Am. 1987 12;82(6):1901–12. 3429729 10.1121/1.395685

[14] Watkins WA, Moore KE, Wartzok D, Johnson JH. Radio tracking of finback (Balaenoptera physalus) and humpback (Megaptera novaeangliae) whales in Prince William Sound, Alaska. Deep Res. 1981;28(6):577–88.

[15] Watkins WA, Moore KE, Sigurjonsson J, Wartzok D, Notobartolo di Sciara G. Fin whale (Balaenoptera physalus) tracked by radio in the Irminger Sea.J Mar Res Inst. 1984;(8):1–14 p.

[16] Castellote M, Clark CW, Lammers MO. Fin whale (Balaenoptera physalus) population identity in the western Mediterranean Sea. Mar Mammal Sci. 2012;28(2):325–44.

[17] Sirović A, Hildebrand JA, Wiggins SM. Blue and fin whale call source levels and propagation range in the Southern Ocean. J Acoust Soc Am. 2007;122(2):1208–15. doi: 10.1121/1.2749452 17672667 10.1121/1.2749452

[18] McDonald MA, Hildebrand JA, Webb SC. Blue and fin whales observed on a seafloor array in the Northeast Pacific. J Acoust Soc Am. 1995;98(2):712–21. 7642810 10.1121/1.413565

[19] Moore SE, Stafford KM, Dahlheim ME, Fox CG, Braham HW, Polovina JJ, et al Seasonal variation in reception of fin whale calls at five geographic areas in the North Pacific. Mar Mammal Sci. 1998;14(3):617–27.

[20] Thompson PO, Findley LT, Vidal O. 20-Hz Pulses and other vocalizations of Fin Whales, Balaenoptera-Physalus, in the Gulf of California, Mexico. J Acoust Soc Am. 1992;92(6):3051–7. 1474220 10.1121/1.404201

[21] Northrop J, Cummings WC, Thompson PO. 20-Hz signals observed in the Central Pacific. J Acoust Soc Am. 1968;43(2):13–4.

[22] Širović A, Hildebrand JA, Wiggins SM, McDonald MA, Moore SE, Thiele D. Seasonality of blue and fin whale calls and the influence of sea ice in the Western Antarctic Peninsula. Deep Sea Res Part II Top Stud Oceanogr. 2004;51(17):2327–44.

[23] Simon M, Stafford KM, Beedholm K, Lee CM, Madsen PT. Singing behavior of fin whales in the Davis Strait with implications for mating, migration and foraging. J Acoust Soc Am. 2010;128(5):3200–10. doi: 10.1121/1.3495946 21110615 10.1121/1.3495946

[24] Nieukirk SL, Mellinger DK, Moore SE, Klinck K, Dziak RP, Goslin J. Sounds from airguns and fin whales recorded in the mid-Atlantic Ocean, 1999–2009. J Acoust Soc Am. 2012;131(2):1102 doi: 10.1121/1.3672648 22352485 10.1121/1.3672648

[25] Croll D a, Clark CW, Acevedo A, Tershy B, Flores S, Gedamke J, et al Only male fin whales sing loud songs. Nature. 2002;417(6891):809. 10.1038/417809a 12075339

[26] Broughton WB. Methods in bio-acoustic terminology. Acoust Behav Anim. 1963;3–24.

[27] Garland EC, Goldizen AW, Rekdahl ML, Constantine R, Garrigue C, Hauser ND, et al Dynamic horizontal cultural transmission of humpback whale song at the ocean basin scale. Curr Biol. 2011;21(8):687–91. doi: 10.1016/j.cub.2011.03.019 21497089 10.1016/j.cub.2011.03.019

[28] Payne K, Payne R. Large scale changes over 19 Years in songs of humpback whales in Bermuda. Z Tierpsychol. 1985;68(2):89–114.

[29] Noad MJ, Cato DH, Bryden MM, Jenner M-N, Jenner KCS. Cultural revolution in whale songs. Nature. 2000;408(6812):537. 10.1038/35046199 11117730

[30] McDonald MA, Mesnick SL, Hildebrand JA. Biogeographic characterisation of blue whale song worldwide: using song to identify populations. J Cetacean Res Manag. 2006;8(1):55–65.

[31] Oleson E, Barlow J, Gordon J, Rankin S, Hildebrand J. Low frequency calls of Bryde’s whales. Mar Mammal Sci. 2003;19(2):407–19.

[32] Rankin S, Barlow J. Source of the North Pacific “boing” sound attributed to minke whales. J Acoust Soc Am. 2005;118(5):3346 16334704 10.1121/1.2046747

[33] McDonald MA, Hildebrand JA, Mesnick S. Worldwide decline in tonal frequencies of blue whale songs. Endanger Species Res. 2009;9:13–21.

[34] Gavrilov AN, McCauley RD, Salgado-Kent C, Tripovich J, Burton C. Vocal characteristics of pygmy blue whales and their change over time. J Acoust Soc Am. 2011;130(6):3651 doi: 10.1121/1.3651817 22225022 10.1121/1.3651817

[35] Delarue J, Todd SK, Van Parijs SM, Di Iorio L. Geographic variation in Northwest Atlantic fin whale (Balaenoptera physalus) song: implications for stock structure assessment. J Acoust Soc Am. 2009;125(3):1774–82. doi: 10.1121/1.3068454 19275334 10.1121/1.3068454

[36] Morano JL, Salisbury DP, Rice AN, Conklin KL, Falk KL, Clark CW. Seasonal and geographical patterns of fin whale song in the western North Atlantic Ocean. J Acoust Soc Am. 2012;132(2):1207 doi: 10.1121/1.4730890 22894239 10.1121/1.4730890

[37] Širović A, Oleson EM, Buccowich J, Rice A, Bayless A. Fin whale song variability in southern California and the Gulf of California. Sci Rep. 2017;in review. 10.1038/s41598-017-09979-4 PMC5579205 28860617

[38] Širović A, Hildebrand JA, Wiggins SM, Thiele D. Blue and fin whale acoustic presence around Antarctica during 2003 and 2004. Mar Mammal Sci. 2009;25(1):125–36.

[39] Gedamke J, Robinson SM. Acoustic survey for marine mammal occurrence and distribution off East Antarctica (30–80°E) in January-February 2006. Deep Sea Res Part II Top Stud Oceanogr. 2010;57(9):968–81.

[40] Delarue J, Martin B, Hannay D, Berchok CL. Acoustic occurrence and affiliation of fin whales detected in the Northeastern Chukchi Sea, July to October 2007–10. Arctic. 2013;66(2):159–72.

[41] Oleson EM, Širović A, Bayless AR, Hildebrand JA. Synchronous seasonal change in fin whale song in the North Pacific. PLoS One. 2014;9(12):1–18. 10.1371/journal.pone.0115678 PMC4270802 25521493

[42] Watkins WA. Activities and underwater sounds of fin whales. Sci Rep Whales Res Inst. 1981;33:83–117.

[43] Širović A, Williams LN, Kerosky SM, Wiggins SM, Hildebrand JA. Temporal separation of two fin whale call types across the eastern North Pacific. Mar Biol. 2013;160(1):47–57. doi: 10.1007/s00227-012-2061-z 24391281 10.1007/s00227-012-2061-z PMC3873066

[44] Soule DC, Wilcock WSD, Thompson RE. Statistical analysis of fin whale vocalizations recorded by a seismic network at the Endeavour Segment of Juan de Fuca Ridge, N. E. Pacific Ocean. J Acoust Soc Am. 2011;129(4):2638.

[45] Cox C, Deaton T, Webb S. A deep-sea differential pressure gauge. J Atmos Oceanogr Technol. 1984;1:237–46.

[46] McGill PR, Wilcock WS, Stakes DS, Barclay AH, Ramirez TM, Toomey DR. A Long-Term Seismic Array on the Endeavour Segment of the Juan de Fuca Ridge. Am Geophys Union, Fall Meet 2003, Abstr #B12A-0748. 2003;

[47] Williams MC, Tréhu AM, Braunmiller J. Seismicity at the Cascadia Plate boundary beneath the Oregon continental chelf. Bull Seismol Soc Am. 2011;101:940–50.

[48] Toomey D, Allen R, Barclay A, Bell S, Bromirski P, Carlson R, et al The Cascadia Initiative: A sea change in seismological studies of subduction zones. Oceanography. 2014;27(2):138–50.

[49] Koot B. Winter behaviour and population structure of fin whales (Balaenoptera physalus) in British Columbia inferred from passive acoustic data University of British Columbia; 2015.

[50] Soule DC, Wilcock WSD. Fin whale tracks recorded by a seismic network on the Juan de Fuca Ridge, Northeast Pacific Ocean. J Acoust Soc Am. 2013;133(3):1751 doi: 10.1121/1.4774275 23464044 10.1121/1.4774275

[51] Stafford KM, Fox CG, Clark CW. Long-range acoustic detection and localization of blue whale calls in the northeast Pacific Ocean. J Acoust Soc Am. 1998;104(6):3616–25. 9857519 10.1121/1.423944

[52] Bracewell RN. The fourier transform and its applications 2nd ed. New York: McGraw-Hill; 1986. 474 p.

[53] Jones E, Oliphant T, Peterson P. SciPy: Open source scientific tools for Python.

[54] Gavrilov AN, McCauley RD, Gedamke J. Steady inter and intra-annual decrease in the vocalization frequency of Antarctic blue whales. J Acoust Soc Am. 2012;131(6):4476–80. doi: 10.1121/1.4707425 22712920 10.1121/1.4707425

[55] Parks SE, Urazghildiiev I, Clark CW. Variability in ambient noise levels and call parameters of North Atlantic right whales in three habitat areas. J Acoust Soc Am. 2009;125(2):1230 doi: 10.1121/1.3050282 19206896 10.1121/1.3050282

[56] Parks SE, Clark CW, Tyack PL. Short- and long-term changes in right whale calling behavior: The potential effects of noise on acoustic communication. J Acoust Soc Am. 2007;122(6):3725 doi: 10.1121/1.2799904 18247780 10.1121/1.2799904

[57] Foote AD, Osborne RW, Hoelzel AR. Whale-call response to masking boat noise. Nature. 2004;428(April):910. 15118717 10.1038/428910a

[58] McKenna MF, Katz SL, Wiggins SM, Ross D, Hildebrand JA. A quieting ocean: Unintended consequence of a fluctuating economy. J Acoust Soc Am. 2012;132:EL169 doi: 10.1121/1.4740225 22979828 10.1121/1.4740225

[59] Andrew RK, Howe BM, Mercer JA. Long-time trends in ship traffic noise for four sites off the North American West Coast. The Journal of the Acoustical Society of America 129:642–651. J Acoust Soc Am. 2011;129:642–51. doi: 10.1121/1.3518770 21361423 10.1121/1.3518770

[60] Hatch LT. Acoustic differentiation among fin whales, Balaenoptera physalus, in the North Atlantic and North Pacific Oceans, and integration with genetic estimates of divergence. In: PhD Thesis. Cornell University; 2004. p. 160–237.

[61] Boyd R, Richerson PJ. Culture and the evolutionary process University of Chicago Press; 1988. 331 p.

[62] Rendell L, Whitehead H. Culture in Whales and Dolphins 292 C Culture in Whales and Dolphins. Mar Ecol Prog Ser. 2007;52(337):175–80.

[63] Deecke VB, Ford JKB, Spong P. Dialect change in resident killer whales: implications for vocal learning and cultural transmission. Anim Behav. 2000;60(5):629–38. doi: 10.1006/anbe.2000.1454 11082233 10.1006/anbe.2000.1454

[64] Cerchio S, Jacobsen JK, Norris TF. Temporal and geographical variation in songs of humpback whales, Megaptera novaeangliae: synchronous change in Hawaiian and Mexican breeding assemblages. Anim Behav. 2001;62(2):313–29.

[65] Gerhardt HC. The Evolution of Vocalization in Frogs and Toads Author (s): H. Carl Gerhardt Source : Annual Review of Ecology and Systematics, Vol. 25 (1994), pp. 293–324 Published by : Annual Reviews Stable URL : http://www.jstor.org/stable/2097314. Annu Rev Ecol Syst. 1994;25(1994):293–324.

[66] Sun K, Luo L, Kimball RT, Wei X, Jin L, Jiang T, et al Geographic variation in the acoustic traits of greater horseshoe bats: Testing the importance of drift and ecological selection in evolutionary processes. PLoS One. 2013;8(8):1–10. 10.1371/journal.pone.0070368 PMC3738568 23950926

[67] Jenkins PF. Cultural transmission of song patterns and dialect development in a free-living bird population. Anim Behav. 1978;26(PART 1):50–78.

[68] Slater PJB. The cultural transmission of bird song. Trends Ecol Evol. 1986;1(4):94–7. doi: 10.1016/0169-5347(86)90032-7 21227788 10.1016/0169-5347(86)90032-7

[69] West MJ, King AP, Harrocks TJ. Cultural transmission of cowbird song (Molothrus ater): Measuring its development and outcome. J Comp Psychol. 1983;97(4):327–37.

[70] Payne R, Webb D. Orientation by means of long range acoustic signaling in baleen whales. Ann N Y Acad Sci. 1971;188:110–41. 5288850 10.1111/j.1749-6632.1971.tb13093.x

[71] Stafford KM, Moore SE. Atypical calling by a blue whale in the Gulf of Alaska (L). J Acoust Soc Am. 2005;117(5):2724 15957742 10.1121/1.1887005

[72] Mizroch S a., Rice DW, Zwiefelhofer D, Waite J, Perryman WL. Distribution and movements of fin whales in the North Pacific Ocean. Mamm Rev. 2009;39(3):193–227.

[73] Bérubé M, Urbán JR, Dizon AE, Brownell RL, Palsbøll PJ. Genetic identification of a small and highly isolated population of fin whales (Balaenoptera physalus) in the Sea of Cortez, México. Fish Sci. 2002;3:183–90.

[74] Fujino K. Immunogenetic and marking approaches to identifying subpopulations of the North Pacific whales. Sci Rep Whales Res Inst, Tokyo. 1960;15:84–142.

[75] Delaney JR, Kelley DS. Next-generation science in the ocean basins: Expanding the oceanographer’s toolbox utilizing submarine electro-optical sensor networks In: Seafloor Observatories. Berlin, Heidelberg: Springer Berlin Heidelberg; 2015 p. 465–502.

[76] Barnes CR, Best MMR, Johnson FR, Pirenne B. NEPTUNE Canada: Installation and initial operation of the world’s first regional cabled ocean observatory In: Seafloor Observatories. Berlin, Heidelberg: Springer Berlin Heidelberg; 2015 p. 415–38.

